# Using climate envelope models to identify potential ecological trajectories on the Kenai Peninsula, Alaska

**DOI:** 10.1371/journal.pone.0208883

**Published:** 2018-12-26

**Authors:** Dawn Robin Magness, John M. Morton

**Affiliations:** Kenai National Wildlife Refuge, U. S. Fish and Wildlife Service, Soldotna, Alaska, United States of America; Michigan State University, UNITED STATES

## Abstract

Managers need information about the vulnerability of historical plant communities, and their potential future conditions, to respond appropriately to landscape change driven by global climate change. We model the climate envelopes of plant communities on the Kenai Peninsula in Southcentral Alaska and forecast to 2020, 2050, and 2080. We assess 6 model outputs representing downscaled climate data from 3 global climate model outputs and 2 representative concentration pathways. We use two lines of evidence, model convergence and empirically measured rates of change, to identify the following plausible ecological trajectories for the peninsula: (1.) alpine tundra and sub-alpine shrub decrease, (2.) perennial snow and ice decrease, (3.) forests remain on the Kenai Lowlands, (4.) the contiguous white-Lutz-Sitka spruce complex declines, and (5.) mixed conifer afforestation occurs along the Gulf of Alaska coast. We suggest that converging models in the context of other lines of evidence is a viable approach to increase certainty for adaptation planning. Extremely dynamic areas with multiple outcomes (i.e., disagreement) among models represent ecological risk, but may also represent opportunities for facilitated adaptation and other managerial approaches to help tip the balance one way or another. By reducing uncertainty, this eclectic approach can be used to inform expectations about the future.

## Introduction

Climate change has the potential to transform ecosystems, create novel species assemblages, and increase extinction rates [1–4]. In order to apply management approaches for adaptation, managers need to understand the vulnerability of current species and habitats including the range of potential future conditions [5–9].

Vulnerability assessment is a framework to evaluate climate change exposure and the characteristics that allow species or ecosystems to absorb or respond to change [10]. Translating vulnerability assessments into knowledge about the range of potential future conditions is challenging because of uncertainty in both the climate projections and ecological responses [11]. Uncertainty in climate projections can be addressed in part by considering a range of emission scenarios, global climate models (GCMs), and downscaling approaches. Uncertainties about ecological responses will likely lead to surprises because ecosystems can reorganize once unknown thresholds are exceeded, novel species assemblages can lead to complex interactions, and species may encounter novel climate conditions including climate extremes [1,2]. Natural resource managers can still move forward by exploring a range of potential future conditions, keeping management responses nimble in order to respond to new information, and strategically coordinating disparate adaptation approaches to bet-hedge and maximize learning [9,12].

Uncertainty about ecological responses does not mean that the best available information is not useful. Natural resource managers benefit from integrating multiple lines of evidence to find where there is agreement among methods, and to visualize or imagine a wider range of future conditions [11]. At regional and landscape scales, sources of information include down-scaled climate data, estimates of climate velocity, climate envelope models, mechanistic vegetation models, paleoecology, and empirical rates of change from 20th century observations [11,13]. Here, we utilize down-scaled climate data, climate envelope models, and empirical rates of change to identify plausible ecological trajectories for a climatically-dynamic Alaskan landscape. We discuss how these potential ecological trajectories can be engaged by natural resource managers to design monitoring and research that can provide additional evidence, and to develop and implement strategically-coordinated adaptation strategies.

## Study area

**Fig 1:**
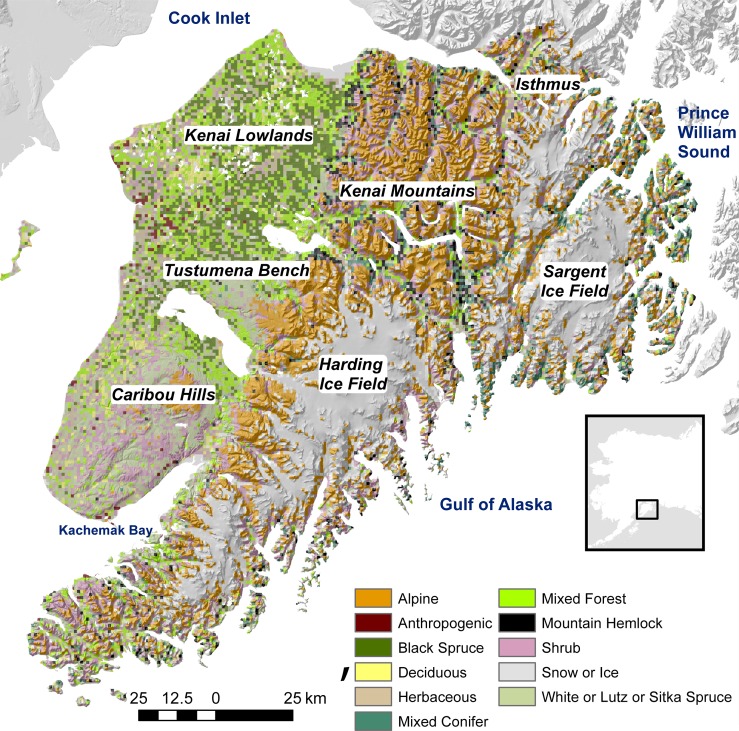
Study area. The Kenai Peninsula in south-central Alaska is separated from the adjacent mainland by a ~16-km wide isthmus. The Kenai Mountains run southwest to northeast. The Kenai Lowlands and areas west of the Kenai Mountains are characterized as boreal forest. The eastern Kenai Peninsula is characterized as Pacific maritime rainforest. Three federal land agencies administer ~75% of the land.

The 24,300 km^2^ Kenai Peninsula juts out into the Gulf of Alaska and is connected to the mainland by a 16-km wide isthmus (Fig 1). Topography is diverse, ranging from sea level to >1,600 m in the Kenai Mountains where three icefields lie: the Harding, Sargent, and Grewingk-Wosnesenski complex. The Kenai Peninsula straddles the southwestern extent of the boreal forest and the northwestern extent of temperate coastal rainforest. Coastal influence and the Kenai Mountains create a rain-shadow effect that increases the range of climatic conditions. A gradient of vegetation communities occurs with black spruce (*Picea mariana*) dominated forests on the northwestern peninsula, changing to white (*P*. *glauca*) and Lutz spruce (*P*. x *lutzii*), and finally to Sitka spruce (*P*. *sitchensis*) to the south and east. Black spruce forests have a 80-year fire return interval leading to a diverse matrix of forest types [14]. White and Lutz spruce have historically been disturbed by 50-year regional outbreaks of spruce beetle (*Dendroctonus rufipennis*) and less frequently by fires and wind [15,16]. Sitka spruce forests are not typically subject to fire, but have historically been disturbed by beetles [17]. Tree line is typically mountain hemlock (*Tsuga mertensiana*), spruce (*Picea spp*.), or alder (*Alnus spp*.).

## Methods

### Climate envelope modelling

We modeled the current and forecast the future climate envelope of vegetation communities across a range of future climates [18]. We used 10 vegetation communities classified from 2002 Landsat-7 ETM imagery (Table 1). Erdas Imagine was used to mosaic 4 satellite images and to run the Tasseled Cap transformation and Normalized Difference Vegetation Index (NDVI). The image mosaic, Tasseled Cap transformation, NDVI, and DEM were segmented into 0.1 ha minimum mapping units using the multi-resolution segmentation routine in eCognition. Field site locations (n = 4,074) were split into training and validation (20%) datasets. The training data were used to build a 26-type class hierarchy. The classification accuracy was 65%. We collapsed the 26 classes into 10 generalized vegetation communities. We rescaled the 0.1 ha vegetation classification to a resolution that matched the climate data by assigning the vegetation community with the largest area in each 1-km climate cell. To understand the overlap of vegetation communities lost by rescaling to the resolution of the climate data, we calculated the average area within each climate cell occupied by the assigned vegetation type and other vegetation types.

**Table 1:** Vegetation communities. Descriptions of the ten vegetation communities found on the Kenai Peninsula, Alaska. Ecological type was derived from a 2002 Landsat classification and used to develop a climate envelope model to forecast future ecological conditions.

Name	Description
Alpine	High elevation areas consisting of tundra, low mat shrubs, sparse vegetation, rock outcrops, and rock exposed from glacial scouring
Black Spruce	Black spruce (*Picea mariana*) dominated forests occurring on the Kenai Lowlands and Tustumena Bench
Deciduous	Hardwood dominated areas consisting of quaking aspen (*Populus tremuloides*), paper birch (*Betula neoalaskan*), black cottonwood (*Populus balsamifera spp*. *trichocarpa*), and balsam poplar (*Populus balsamifera spp*. *balsamiferma*)
Herbaceous	Low elevation, dry to wet graminoid-herbaceous dominated areas. Upland and dry sites generally include bluejoint reedgrass (*Calamagrostis canadensis*), red fescue (*Festuca rubra*), Altai fescue (*Festuca altaica*), lupine (*Lupinus spp*.), fireweed (*Epilobium angustifolium*), and cow parsnip (*Heracleum lanatum*). Moderately wet sites shift to sedges (*Carex spp*.) and wet sites to cottongrass (*Eriophorum spp*.) and buckbean (*Menyanthes trifoliate*)
Ice	High elevation perennial snow and ice
Mixed Conifer	Closed forest dominated by mountain hemlock (*Tsuga mertensiana*) and Sitka spruce (*Picea sitchensis*) generally occurring along Prince William Sound in the eastern Kenai Peninsula.
Mixed Forest	Intermixed hardwood and softwood tree species such as spruce, quaking aspen, paper birch and cottonwoods generally occurring at low elevations from the Kenai Lowlands to south of Tustumena Lake.
Mountain Hemlock	Mountain hemlock dominated forest generally occurring around Seward, in isolated areas of the Kenai Mountains, and along the coast.
Shrub	Isolated low elevation, coastal and riparian areas dominated by alder (*Alnus spp*.), willow (*Salix spp*.), other shrubs such as salmonberry (*Rubus spectabilis*), mountain-ash (*Sorbus sitchensis*) and *Vaccinium spp*. and wetland shrubs such as Labrador tea (*Ledum palustre*) and dwarf birch (*Betula nana*).
White-Sitka Spruce	Forest dominated by white spruce, Sitka spruce, and Lutz spruce (*Pices x lutzii [glauca x sitchensis]*), a white-Sitka hybrid, that generally occurs in the eastern foothills of the Kenai Mountains, the Tustumena Bench, surrounding the Caribou Hills, and south of Katchemak Bay. White spruce is more common in the northern lowlands and Sitka spruce is more common in southern coastal areas around Homer and on the eastern Kenai Peninsula.

We downloaded AdaptWest (http://adaptwest.databasin.org/pages/adaptwest-climatena) climate surfaces to use as predictors [12] (Table 2). We used the Random Forest (Salford Systems; www.salford-systems.com) algorithm to build climate envelope models for the 1960–1990 baseline and to forecast vegetation in the 2020s, 2050s, and 2080s under a range of GCMs and emission scenarios. We used all 23 climate variables even though some were correlated >0.7 in the baseline data because Random Forest does not require noncollinearity and the correlations changed when the climate variables were forecast to future conditions [19]. The Random Forest algorithm produces accurate predictions and has been widely used for ecological applications [18,20–22]. When compared to other algorithms, Random Forest performs extremely well in applications where extrapolating to future conditions is the goal [23]. Random Forest builds multiple regression trees (n = 200) and averages the trees to maximize predictive ability [19]. For each tree, we excluded 20% of the data (out-of-bag data) to be used to assess model error. We chose 3 GCM outputs to represent the highest climate change exposure for Alaska (GFDL-CM3), the lowest exposure (INM-CM4), and mid-level exposure (average of 15-GCMs). We considered 2 representative concentration pathways (RPCs) for each GCM: RPC 45 and RPC 85. RPCs are storylines of future conditions that include information about emissions, gas concentrations, and land-use trajectories [24]. RCPs differ in terms of the amount of radiative forcing (higher radiative forcing leads to higher climate change exposure). RCP 85 is the high radiative forcing pathway (8.5 W/m^2^ by 2100) whereas RCP 45 represents a future pathway where radiative forcing stabilizes without overshoot to 4.5 W/m^2^) after 2100.

**Table 2:** Bioclimatic variables. Twenty-three ecologically relevant bioclimatic variables provided by Adaptwest (http://adaptwest.databasin.org/pages/adaptwest-climatena). These variables were used as predictors to build climate envelope models of Kenai Peninsula vegetation communities.

Bioclimatic Variables
mean annual temperature (°C)
mean temperature of the warmest month (°C)
mean temperature of the coldest month (°C)
difference between coldest and warmest month—measure of continentality (°C)
mean annual precipitation (mm)
mean summer (May to Sep) precipitation (mm)
annual heat moisture index
degree-days below 0°C
degree-days above 5°C
degree-days below 18°C
degree-days above 18°C
number of frost-free days
Julian date on which the frost-free period begins
Julian date on which the frost-free period ends
frost-free period
precipitation as snow (mm)
extreme minimum temperature over 30 years
extreme maximum temperature over 30 years
Hargreave's reference evaporation
Hargreave's climatic moisture index
mean annual solar radiation
mean annual relative humidity (%)

We calculated the mean values of the 4 bioclimatic variables with the highest importance rankings in the Random Forest output. The mean represents the average value of the downscaled climate surface on the Kenai Peninsula. We summarized the bioclimatic variables by vegetation community for the baseline climate (1960–1990) and across the 6 forecasts (3GCMs x 2 RCPs) for 2080.

### Identifying robust ecological trajectories

We calculated the minimum and maximum area of each vegetation community in 2080 for the 6 model outputs (3 GCMs x 2 RPCs). We compared the 2080 area with the area currently occupied by each vegetation community to assess agreement about the trajectory of climate space for the vegetation type.

We mapped the forecasted vegetation community across the 6 model outputs by averaging the Random Forest value (range 0–1) for each vegetation community at each pixel in the 2020s, 2050s, and 2080s. We assigned the vegetation community with the highest average value. We mapped the vegetation community forecast by each of the 6 model outputs in 2080s to visualize the range of outputs. To assess uncertainty, we mapped the number of model outputs (range 1–6) that converged on the same vegetation community and the diversity of vegetation communities forecast. We mapped the number of vegetation conversions between time steps to index temporal stability [25].

For this analysis, we forecasted the vegetation type using maximum relative likelihood value. For comparison, we also applied a threshold to mediate change when mapping the forecasted vegetation. Ecosystems can rapidly restructure after disturbance [1]. Prior to disturbance, the status quo can be maintained even as key structuring conditions have changed. After disturbance, the species and seed sources available will determine the ecological trajectory [26]. The ecological legacies (i.e., soil conditions, seed bank, and dispersal from undisturbed areas) reinforce re-establishment of the previous vegetation type as long as the climatic niche has not radically shifted. For this relative comparison of unlimited change and change mediated by legacy, we used a threshold of 0.1 because there are 10 land cover types which split the relative likelihood that sums to one. If the historical vegetation community maintained >0.1 maximum relative likelihood value with the forecasted future climate data, we maintained the historical vegetation type even when another type had a higher relative likelihood.

Finally, we conducted a literature review of climate change effects that are based on empirical observations from the Kenai Peninsula. We compared the empirical trends for the Kenai Peninsula with the ecological trajectories identified in the climate envelope models.

## Results

### Climate envelope model assessment

The climate envelope model had a 59% misclassification rate when predicting the current vegetation type of 1-km climate cells. Random assignment of vegetation type results in a 90% misclassification error. The prediction accuracy of the climate envelope model ranged from 23–80% depending on vegetation type (Table 3). Before rescaling the vegetation, more than one vegetation type occupied the 1-km climate cell. On average, the assigned vegetation type covered 36–84% of the 1-km climate cell (Table 3). Prediction accuracy of the climate envelope model is higher for vegetation types that are more contiguous and therefore, were more likely to occupy a large area of 1-km climate cell. On average, snow or ice occupied 84% of the area of climate cells that were classified as snow or ice and had the highest prediction accuracy. Deciduous, mountain hemlock and herbaceous vegetation types were highly interspersed with other vegetation types within each 1-km climate cell. Shrub vegetation had low classification accuracy and was highly interspersed with 6 other vegetation types. Shrub was often misclassified as alpine, which is likely related to the occurrence of sub-alpine shrub in an elevational band below alpine on the Kenai Peninsula. Mixed forest also had low classification accuracy with the climate envelope model. Mixed forest co-occurs with black spruce within the 1-km climate cell and the climate envelope model often misclassified mixed forest cells as black spruce forest. The mixed forest type represents a blend of coniferous and deciduous forest types and therefore may not be distinguishable from spruce and deciduous types by climate.

**Table 3:** Model prediction accuracy. Vegetation types on the Kenai Peninsula, Alaska, were classified at the 0.1 ha scale and rescaled to match the resolution of climate surface data for climate envelope modelling. Prediction accuracy for each vegetation type is based on the ability of the climate envelope model to predict the current vegetation type of each climate cell. We assigned each climate cell the vegetation community with the largest area. Percent area is the average area of the 1-km climate cell occupied by the assigned vegetation type prior to rescaling the vegetation. We identify vegetation types as co-occurring when they occupy ≥ 10% of the climate cell area. Co-occurring vegetation identifies vegetation types that occur within climate cells assigned to another type and the average percent of the 1-km cell occupied by the unassigned type. We identify the vegetation types that are most likely to be misclassified as another type by the climate envelope model as vegetation types that represent ≥ 20% the misclassified cells.

	Prediction Accuracy	Percent Area	Co-occurring Vegetation Communities (Percent Area)	Mostly Misclassified As (Percent of Misclassifications)
**Alpine**	48%	63%	Snow/Ice (14%), Shrub (13%)	Snow/Ice (28%), Shrub (23%), Mixed Conifer (20%)
**Black Spruce**	50%	54%	Mixed Forest (17%)	Mixed Forest (31%), Herbaceous (27%), Deciduous (24%)
**Deciduous**	35%	36%	Mixed Forest (18%), Shrub (12%),	Mixed Forest (22%)
**Herbaceous**	25%	36%	Shrub (13%), Black Spruce (11%),	Black Spruce (22%)
**Mixed Conifer**	56%	46%	Alpine (16%), Shrub (11%)	Mountain Hemlock (38%)
**Mixed Forest**	23%	49%	Black Spruce (11%), White-Lutz-Sitka Spruce (11%)	Black Spruce (28%),
**Mountain Hemlock**	36%	36%	Shrub (13%), Alpine (11%), Mixed Conifer (10%)	Mixed Conifer (28%),
**Shrub**	23%	49%	Alpine (19%)	Alpine (21%),
**Snow or Ice**	80%	84%	Alpine (12%)	Alpine (64%)
**White-Lutz-Sitka Spruce**	32%	52%	Shrub (13%), Mixed Forest (12%)	No type accounts for >20% of error

### Climate exposure by vegetation community

Regional climate trends were stable across all forecasts for the 4 most important variables in the climate envelope model (Table 4). Mean annual precipitation and temperature are forecasted to increase. Precipitation and temperature interact, leading to an increase in the annual heat moisture index and Hargreave’s reference evaporation. Precipitation as snow is forecasted to decrease.

**Table 4:** Climate variable summary. Average values of Adaptwest baseline (1960–1990) climate for the Kenai Peninsula, Alaska. We summarized annual heat moisture index, precipitation as snow, mean annual precipitation, and Hargreve’s reference evapotranspiration because these are the 4 highest variables ranked for importance in the Random Forest model. We summarized mean annual temperature because temperature interacts with precipitation to calculate the annual heat moisture index and Hargreaves reference evaporation. We calculate the percent change from baseline for the 6 future climate forecasts using 3 GCMs and 2 RCPs in 2080.

	Annual Heat Moisture Index (mean annual temperature+10)/ (mean annual precipitation/1000)	Precipitation as Snow (mm)	Mean Annual Precipitation (mm)	Hargreave's Reference Evaporation	Mean Annual Temperature (°C)
Baseline	12	862	1735	351	1.9
INM-CM4 RCP45	10%	-27%	9%	7%	113%
15-model Average RCP45	16%	-42%	14%	19%	193%
INM-CM4 RCP85	17%	-59%	18%	17%	237%
15-model Average RCP85	22%	-69%	25%	31%	315%
GFDL-CM3 RCP45	23%	-60%	17%	29%	269%
GFDL-CM3 RCP85	26%	-76%	28%	37%	365%

Moisture availability is an important driver of vegetation communities on the Kenai Peninsula. The variable importance ranking identified the 4 most important variables for prediction accuracy to be annual heat moisture index, precipitation as snow, mean annual precipitation, and Hargreave’s reference evapotranspiration. Precipitation as snow ranged from 213–1751 mm with the black spruce vegetation community having the lowest value and perennial ice and snow having the highest value (Table 5). Black spruce, deciduous and mixed forests occupy areas with the least moisture available. Black spruce is limited climatically to areas with low moisture availability that coincide with poorly-drained soils of the glacial moraine complex on the northwestern Kenai Peninsula (i.e., Kenai Lowlands). The white-Lutz-Sitka spruce complex and herbaceous vegetation communities require more moisture than black spruce while deciduous and mixed forest occupy a wider moisture gradient. Alpine, mixed conifer and snow occupy the wettest areas. There is significant overlap in the climate across vegetation types. The climate envelope model only considers how climate constrains vegetation communities, but other factors, such as disturbance history and soil also influence vegetation distributions.

**Table 5:** Climate by vegetation. Mean and range of Adaptwest baseline (1960–1990) climate summarized by vegetation community for the Kenai Peninsula, Alaska. Percent change in climate variables by vegetation community in 2080 as compared to the 1960–1990 baseline. The 2080s values are the means for areas forecasted to be a vegetation community under theINM-CM4 RCP (lowest exposure) and GFDL-CM3 RCP85 (highest exposure) scenarios.

	Annual Heat Moisture Index (mean annual temperature+10)/ (mean annual precipitation/1000)		Precipitation as Snow (mm)		Mean Annual Precipitation (mm)		Hargreave's Reference Evaporation		Mean Annual Temperature (°C)	
	Baseline Mean (Range)	2080 INM RCP45 / GFDL RCP 85	Baseline Mean (Range)	2080 INM RCP45 / GFDL RCP 85	Baseline Mean (Range)	2080 INM RCP45 / GFDL RCP 85	Baseline Mean (Range)	2080 INM RCP45 / GFDL RCP 85	Baseline Mean (Range)	2080 INM RCP45 / GFDL RCP 85
Alpine	8 (1.1–25.6)	12 / na	960.7 (178–4782)	727 / na	1936 (451–6424)	2180 / na	332 (209–424)	368 / na	1.8 (-2.7–5.1)	3.9 / na
Black Spruce	24 (3.4–30.6)	4 / 13	212.4 (141–1920)	1181 / 188	519 (408–3471)	3803 / 2661	415 (328–443)	338 / 484	1.9 (0.5–4.1)	4.2 / 9.1
Deciduous	21 (3.5–30.4)	14 / 16	330.7 (140–1817)	547 / 178	751 (409–3805)	1805 / 2117	393 (273–443)	379 / 496	2.2 (-0.1–4.7)	4.2 / 9.0
Herbaceous	18 (2.6–30.4)	14 / 15	457.1 (141–2312)	560 / 228	1127 (409–4611)	1717 / 2182	384 (250–442)	380 / 479	2.3 (-1.2–5.2)	4.1 / 8.7
Mixed Conifer	7 (2.4–36.4)	12 / 14	937.5 (133–2500)	680 / 227	2584 (339–4946)	1893 / 2420	348.9 (242–425)	371 / 474	3.2 (-1.6–5.2)	3.9 / 8.8
Mixed Forest	20 (2.4–30.5)	12 / na	345.3 (140–2424)	419 / na	817 (409–5017)	1141 / na	395.5 (272–443)	367 / na	2.2 (-0.1–5.2)	4 / na
Mountain Hemlock	11 (2.7–27.6)	12 / 12	684.4 (162–2345)	639 / 221	1540 (435–4522)	1928 / 2285	359.2 (246–431)	374 / 465	2.3 (-1.6–5.2)	4.1 / 8.9
Shrub	12 (1.6–29.9)	na / na	660 (143–3682)	na / na	1413 (412–5103)	na / na	356.1 (233–440)	na / na	2.2 (-2.1–5.1)	na / na
Snow or Ice	4 (1.4–16.1)	12 / na	1751.6 (381–4121)	737 / na	3127 (752–5971)	2211 / na	297.5 (204–396)	369 / na	1.1 (-2.9–4.7)	4.0 / na
White—Stika Spruce	17 (2.6–30.4)	29 / na	399.3 (141–2363)	134 / na	892 (395–4560)	514 / na	377.4 (250–442)	450 / na	2.2 (-1.2–5.2)	4.9 / na

In the future outputs, the annual heat moisture index, precipitation as snow, mean annual precipitation, and Hargreave’s reference evapotranspiration were all maintained within the historic range for forecasted vegetation communities (Table 5). Mean annual temperature was above the historic baseline range.

### Identifying plausible ecological trajectories

Most vegetation communities had a convergent trend across all model outputs. Alpine, mixed forest, shrub, the white-Lutz-Sitka spruce complex, and perennial ice or snow decreased in all models (Table 6). Deciduous, herbaceous, mixed conifer and mountain hemlock increased in all model outputs. Black spruce did not have a consistent trend across model outputs.

**Table 6:** Ecological trajectories. Mean, minimum and maximum hectares of each vegetation community forecast by the climate envelope model across 6 model outputs on the Kenai Peninsula, Alaska. Model outputs vary across 2 RCPs and 3GCMs. Trends are assessed by comparing the range with the hectares from the 1km^2^ vegetation.

	1960–1990 Average (Ha)	2080 Average (Ha)	2080 Minimum (Ha)	2080 Maximum (Ha)	Trend	Empirical Evidence
Alpine	432,000	1,750	0	8,500	**-**	- 1.2 m/yr tundra lost to treeline rise [27]; +5%/decade increase in woody vegetation [28]
Black Spruce	216,300	103,950	2,400	256,300	**unk**	Increasing woodiness of wetlands [29]
Deciduous	131,400	628,267	173,200	754,900	**+**	N/A
Herbaceous	167,000	716,333	168,300	1,058,500	**+**	Increasing grass cover in disturbed areas [30]
Mixed Conifer	157,600	613,917	198,800	890,100	**+**	+ 0.1–1.1 m/yr into alpine [27]; +14%/decade into alpine [28]
Mixed Forest	236,400	0	0	0	**-**	N/A
Mountain Hemlock	114,400	369,917	264,200	490,700	**+**	+ 0.1–1.1 m/yr into alpine [27]; +14%/decade into alpine [28]
Shrub	346,300	0	0	0	**-**	+ 2.8 m/yr into alpine [27]; +4%/decade in alpine [28]; increasing ericaceous shrub colonizing peatlands [31]
Snow or Ice	486,500	58,267	0	218,700	**-**	- 5% surface area from 1950 [32]; -21 m elevation [33]; -0.74 - -0.47 m/yr glacial thinning [34]
White-Sitka-Lutz Spruce	243,500	0	0	0	**-**	- 87% basal area and reduction in seedling recruitment [30]

**Fig 2:**
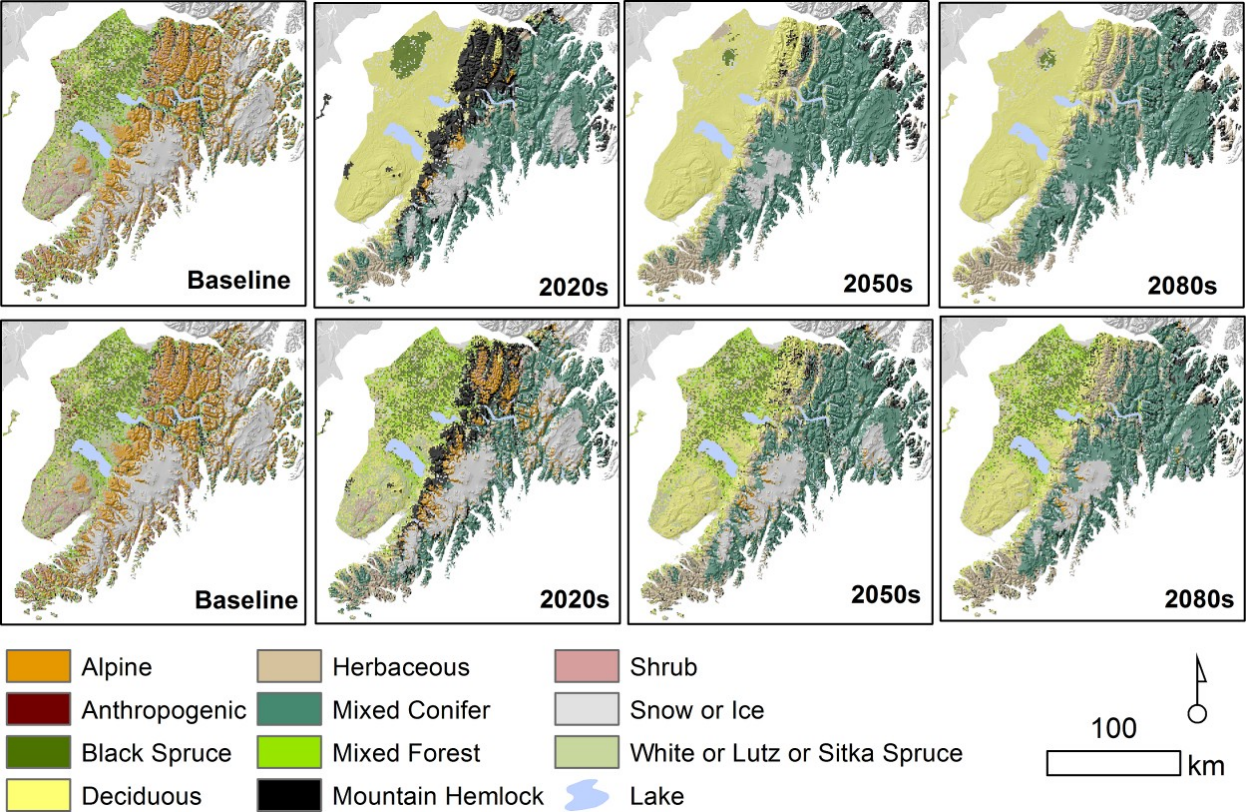
Baseline and forecast vegetation. Comparison of the baseline vegetation on the Kenai Peninsula, Alaska, and the 2020, 2050 and 2080 forecasts. The forecasts represent the vegetation with the highest 6-model average score (top row) and the highest average score moderated by a minimum threshold for change (lower row). The 6-model average represents 2 RCPs and 3 GCMS.

Across model outputs, general trends included loss of alpine above treeline to mountain hemlock, mixed conifer and herbaceous climate envelopes (Fig 2). We also found a general trend of afforestation by coastal rainforest on the eastern Kenai Peninsula. White, Lutz or Sitka spruce and mixed forest vegetation communities in the southeastern Kenai Peninsula converted to deciduous forest. The northern Kenai Lowlands remained forested, in some models outputs black spruce remained while in others, deciduous forest increased.

**Fig 3:**
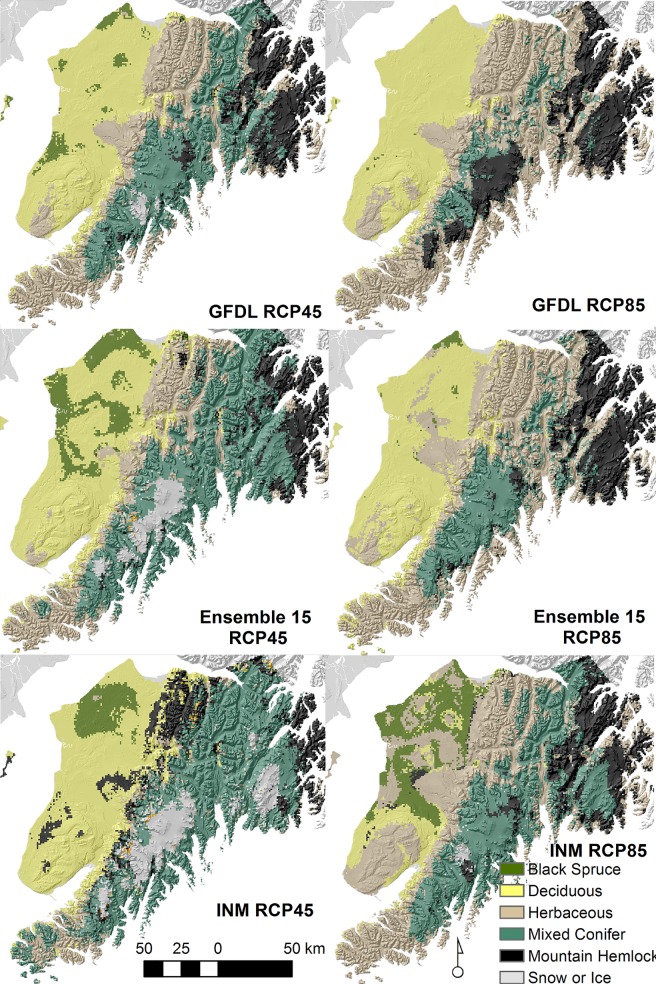
Range of model outputs. Six maps of the Kenai Peninsula representing model outputs for 3 GCMs and 2 RCPs in 2080. GFDL is the high exposure GCM, the Ensemble is mid-level exposure GCM, and INM is the low exposure GCM. RCP 45 is the lower exposure, stabilized radiative forcing emission scenario and RCP 85 is the higher exposure, high radiative forcing scenario.

**Fig 4:**
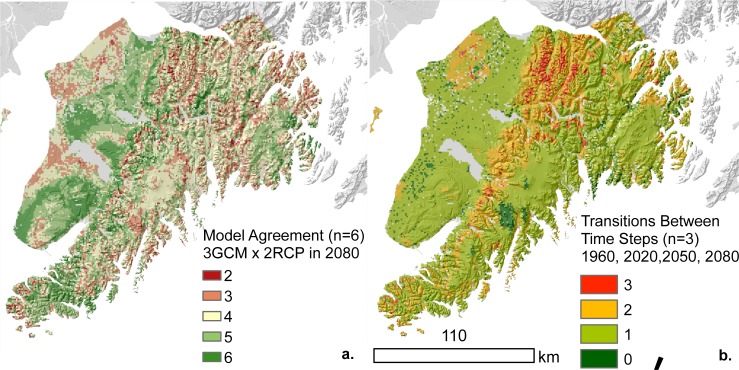
Mapping uncertainty. Maps visualizing uncertainty in future forecasts for the Kenai Peninsula, Alaska. Model agreement represents the number of models representing 3 GCMS and 2 RCPs that converge on the same vegetation community in the 2080s (a). Transitions between time steps represents the number transitions between the baseline, 2020s, 2050s, and 2080s (b).

Across all models, portions of the Harding Icefield remained stable (Fig 3). In contrast, alpine and subalpine areas in the Kenai Mountains and Caribou Hills were consistently transitional (Fig 4). The white-Lutz-Sitka spruce complex that occurs north of the city of Homer was also transitional in all models. However, different patterns emerged when model agreement about the ecological conversion pathway was considered (Fig 4). Mixed conifer afforestation along the southeastern coast and in alpine tundra was a very consistent ecological pathway among all models. Divergent ecological pathways occurred for subalpine and alpine vegetation communities on western slopes and in the white-Lutz-Sitka spruce complex north of Homer.

We found 9 peer-reviewed articles that included empirical rates of landscape-scale change on the Kenai Peninsula (Table 6). We summarized the trend and rate by vegetation category. There was empirical evidence that supported the trends for alpine, snow, white-Lutz-Sitka spruce, mountain hemlock and herbaceous vegetation. There were no studies available to support the trends for deciduous and mixed forest. The empirical evidence contradicted the trend of shrub loss in the climate envelope model outputs.

## Discussion

We developed climate envelope models to forecast potential vegetation communities on the Kenai Peninsula. Plausible ecological trajectories were identified when convergent directional signals in the climate envelope model outputs (based on future vegetation area) were supported by empirical data. Other trends were identified, but are more uncertain because they are not supported by empirical evidence or the model outputs were variable across GCMs and emission scenarios. Ecological trajectories, from most to least plausible, are (1.) alpine tundra decreases; (2.) perennial snow and ice decreases; (3.) forests remain on the Kenai Lowlands, though it is unclear how the composition of softwood versus hardwood will change; (4.) the contiguous white-Lutz-Sitka spruce complex declines; and (5.) afforestation of the southeastern coast.

### Plausible to uncertain trends: The evidence

Integrating multiple lines of evidence can help managers understand ecological trends, highlight dynamic or uncertain outcomes, and open a wider range of future conditions for consideration [11]. Here, we compile information about climate envelope model trends in context with observed 20th century rates of change. We also use information from other published models and anecdotal observations to contextualize trends. Our intention is not to use the climate envelope model to predict the future. Rather, we use the models to open lines of inquiry about the range of potential futures and explore where management could effectively shape conditions.

#### Alpine tundra decreases

The signal direction of the climate envelope models converge on a trend of alpine loss, though the model outputs include both afforestation and conversion to herbaceous vegetation. Alpine tundra decline is supported by empirical evidence of tree-line rise. On the Kenai Peninsula, tree line has advanced 10m/decade on cool, north aspect slopes [28] and shrubs have advanced upslope 2.8m/yr [27]. Approximately 29% of the forest-tundra ecotone increased in woodiness in the Kenai Mountains over the past 50 years with closed canopy forest expanding 14%/decade and tall shrub increasing 4%/decade [28]. Throughout Alaska, trees and shrubs have advanced into tundra and increased in cover as the climate warmed over the last century [35]. Paleoecology studies also document that historic warming in Alaska resulted in tree line advance into tundra, though the rate of recruitment was spatially and temporally variable [36].

Biome-scale climate envelope models developed by other researchers also converge on the loss of alpine tundra. Alpine habitats on the Kenai Peninsula in contemporary climate conditions shifted to a climate more similar to coastal hemlock forest by 2060 using a climate envelope model for 46 North American biomes [37]. Mechanistic modeling conducted for other regions in Alaska support the trajectory of alpine loss to afforestation or conversion to grasslands in shifting climate conditions. A simulation of arctic tree-line advance in response to changing temperature, precipitation, and fire regimes suggests that tundra converts when average summer temperatures exceed 10°C, but conversion pathways were variable [38]; whether tundra converted to evergreen or deciduous forest depended on multiple factors beyond climate. When drier summers were simulated, a novel grassland-steppe habitat became substantial. Another mechanistic model that simulated the response of sub-arctic vegetation in northern Alaska also supports tundra loss in warming climates with the ecological trajectory being driven by more complex interactions among dispersal barriers, climate, and disturbance [39].

#### Loss of perennial snow and ice

Our climate envelope models converge on decreasing perennial snow and ice. The models trend toward afforestation, but we presume that bare rock or glacial moraines will dominate these locations given geologic time-lag in the relative near-term. Loss of perennial snow and ice has been documented on the Kenai Peninsula. The Harding Icefield decreased 5% in surface area from 1950 to 1985 [32] and 21 m in average elevation [33]. Glacial fronts have generally been receding on the Kenai Peninsula since the 1980s [40]. Glacial thinning on the Kenai Peninsula accelerated to -0.72 m/yr in the 1990s from -0.47 m/yr from 1950 to 1990 [34]. Recent warming has resulted in reduced glacial ice mass throughout Alaska [35]. Globally, there has been a mass loss of glacial ice that has accelerated in the late 20th century, with Alaska experiencing more ice loss than other regions [41].

#### Forests remain on the Kenai Lowlands

Our climate envelope models suggest that the Kenai Lowlands will remain forested, though it is unclear how the composition will change in terms of the relative abundance of softwood versus hardwood. Since 1968, available water on the Kenai Lowlands decreased 55% due to warming summer temperatures, increased evapotransportation, and lower annual precipitation [31]. Contemporary drying has accelerated afforestation of peatlands post-Pleistocene. Coring samples indicate that black spruce began colonizing 8000-year-old peatlands in the 1850s at the end of the Little Ice Age with encroachment of ericaceous shrubs since the 1970s [31]. The Kenai Lowlands have become woodier as forest cover has expanded into previously open and wet areas that have dried at accelerating rates over the past 50 years [29]. Bark beetles have not caused significant deforestation in the northern Kenai Lowlands; although white spruce was thinned, black spruce and deciduous tree species were not affected [42]. Mixed forest stands shift toward deciduous vegetation as mature white spruce experience mortality and in response to intense fires that burn down to mineral soil. Additionally, lightning strikes have increased on the Kenai Peninsula in the last two decades, consistent with a warmer atmosphere [43].

Climate envelope models at the biome scale provide mixed support for the Lowlands remaining forested. When North American biomes were modeled, the climate niche associated with the Kenai Lowlands remains forest but shifted from an Alaska subarctic conifer forest to a climate more similar to Rocky Mountain montane conifer forest and Rocky Mountain subalpine conifer forest by 2060 [37]. In contrast, other biome-scale models forecast the Kenai Lowlands to be more similar climatically to non-forested biomes by the end of this century. When Alaska and Canada are considered, the climate conditions shift from being consistent with boreal forest to conditions more similar to Saskatchewan prairie and grassland [44]. When only Alaskan biomes are considered, the lowlands are forecast to shift from boreal forest to climate conditions more similar to the Aleutian Islands in 2099 [45]. The Aleutian Island biome is currently distributed from the northern edge of the Alaska Peninsula down the Aleutian Island chain and characterized by cool, moist harsh weather with moist tundra meadows dominated by grass, sedge, and scattered shrub. Mechanistic modelling supports the persistence of forests on the Kenai Lowlands. ALFRESCO is a process-based model that links climate change, fire regimes, and ecological change [46]. On the Kenai Peninsula, ALFRESCO model outputs anticipate increasing landscape flammability in the next century. ALFRESCO forecasts that coniferous forests will remain dominant on the Kenai Peninsula with deciduous ecological types increasing with increasing fire occurrence and intensity [47].

#### White-Lutz-Sitka spruce complex deforestation

In our climate envelope models, the historically more contiguous white-Lutz-Sitka spruce complex north of the city of Homer, the Caribou Hills, and on the Tustumena Bench declines with some uncertainty about whether conversion to deciduous forest or herbaceous cover is most likely. Empirical and anecdotal evidence support the trajectory of deciduous or herbaceous cover replacing the white-Lutz-Sitka spruce complex surrounding the Caribou Hills. On the Kenai Peninsula, nearly 1 million acres of white and Lutz spruce stands were killed by an unprecedented spruce beetle outbreak beginning in the 1980s [17]. Historically, regionally-scaled bark beetle outbreaks occurred approximately every 52 years on the Kenai Peninsula, with beetle eruptions being triggered by 2 consecutive years of above-average summer temperatures [17]. As the climate warms, chronic beetle exposure is anticipated; white and Lutz spruce stands are unlikely to reach old-growth structural characteristics in a warming climate [17].

Ecological shifts are more likely to occur after a disturbance event like the regional bark beetle outbreak [1]. The white-Lutz-Sitka spruce complex had high tree mortality. Basal area decreased by 87% and bluejoint reedgrass (*Calamagrostis canadensis*) increased as it was released from shading. Seedling recruitment for white and Lutz spruce decreased significantly when bluejoint reedgrass litter exceeded a threshold of 60% [30]. Unprecedented spruce mortality in the aftermath of beetle attack, coupled with human-caused grassland fires in spring, have promoted extensive grasslands on the northwestern slope of the Caribou Hills [48]. In fact, it was this qualitative shift in fire regime from canopy fires in late summer to grass fires in spring that prompted the Alaska Division of Forestry to change the official start of the statewide fire season from 1 May to 1 April in 2006. However, it is unclear if this is a successional phase, an ecological shift to savannah grassland, or perhaps a manifestation of the warm phase of the Pacific Decadal Oscillation.

The biome-scale climate envelope models also forecast the white-Lutz-Sitka spruce dominated area on the Kenai Peninsula to have climate conditions more similar to the non-forested Aleutian Island biome or Saskatchewan prairie [44, 45]. The climate signal that corresponds with Saskatchewan prairie and grassland is characterized by hot spring, summer and fall with moderate precipitation [44].

#### Coastal afforestation

Little empirical evidence is available to support the trajectory of coastal afforestation. The Kenai Peninsula sits at the northwestern boundary of the Pacific coastal rainforest. Conceptually, coastal afforestation is consistent with the expectation that species move northward with a warming climate [49]. The biome-scale climate envelope models agree that future climate will remain similar to current conditions for the Pacific Coastal Rainforest biome, but do not account for the redistribution of ecological types within the biome [37,44,45].

### Climate envelope models: Uncertainty, limitations and constraints

Climate envelope models have been widely critiqued and their assumptions and limitations have been covered elsewhere [50–53]. For climate change assessment, assuming a stable ecological niche is problematic because species may experience novel climate conditions and species may have surprising responses [2,54]. Regions with sparse weather stations include more uncertainty because downscaled climate surfaces have more error when extrapolated into unsampled areas [55]. Even with these limitations, comparing the current and range of future climatic envelopes can inform management actions aimed at matching suites of species to emerging climatic conditions and provide an indication of future trends [18].

Climate envelope models are grounded assumptions about the ecological niche but these models are usually applied to single species [50]. In the past, species have responded individually to changing climatic conditions and ecological communities are not expected to remain as a cohesive unit [49,56]. Climate envelope models for plant communities can still be instructive because climate is a key driver of ecological organization [18,37]. Ecosystems with similar climates have similar structure and ecological function despite divergent histories and species compositions [57]. Climate envelope models associate vegetation patterns with historical climate and forecasted vegetation can be thought of as an equilibrium state that the site would eventually transition to given time [58]. We recognize that climate envelope models may not necessarily reflect realized niches because of constraints imposed by edaphic factors or migration barriers or novel competitive interactions but they offer testable trajectories for the purpose of adaptation planning [11,50].

### Implications for adaptation planning

Spatially-explicit forecasts of future ecological trajectories in response to a changing climate are a powerful way of conveying to both land managers and lay persons that directional change should be an expectation. Changing expectations about the future is an opportunity to rapidly transform natural resource management policies and direction [8,59]. Natural resource managers on the Kenai Peninsula will need to consider whether their goals should be retrospective (historically-based) or prospective (future-based) [12]. Prospective management will require both passive approaches such as designating movement corridors or conserving refugia [5,7, 60], defined as stable plant communities, and active facilitation of ecological conditions such as translocating species to novel locales [61], manipulating habitats towards future conditions to prevent phenological and/or trophic mismatch [12], or facilitating ecological transformation [9]. Empirical confirmation of modeled refugia, and consideration of their juxtaposition at varying spatial scales, will be a critical component of progressive land management strategies. Climate refugia can serve as *in situ* seedbanks, population sources, and stepping stones for populating highly transitional areas [45,62]. Extremely dynamic areas with multiple outcomes (i.e., disagreement) among models are ecologically at risk, but may also represent opportunities for facilitated adaptation and other creative approaches to help tip the balance one way or another. The uncertainty and ecological risk inherent in managing towards future conditions can be minimized by testing hypothesized trajectories against current empirical evidence. This eclectic approach of embracing multiple models and lines of evidence encourages the development of multiple hypotheses for focused monitoring and research, a prerequisite to adaptive management.

## Supporting information

S1 Table KP_Landcover_2020_AverageRFscores.csv. Table of cell locations on the Kenai Peninsula, Alaska with Random Forest outputs forecast to 2020 climate conditions under RCP45 and RCP85.
(CSV)

S2 Table KP_Landcover_2050_AverageRFscores.csv. Table of cell locations on the Kenai Peninsula, Alaska with Random Forest outputs forecast to 2050 climate conditions under RCP45 and RCP85.
(CSV)

S3 Table KP_Landcover_2080_AverageRFscores.csv. Table of cell locations on the Kenai Peninsula, Alaska with Random Forest outputs forecast to 2080 climate conditions under RCP45 and RCP85.
(CSV)

S4 Table KP_Landcover_2080_Forecast.csv. Table of cell locations on the Kenai Peninsula, Alaska with forecast vegetation community based on 2080 climate conditions using 3 GCMs and 2 RCPs.
(CSV)
